# Enhancing the Uptake of Earth Observation Products and Services in Africa Through a Multi-level Transdisciplinary Approach

**DOI:** 10.1007/s10712-022-09724-1

**Published:** 2022-08-23

**Authors:** Chinwe Ifejika Speranza, Felicia Olufunmilayo Akinyemi, David Baratoux, Jérôme Benveniste, Natalie Ceperley, Fatima Driouech, Jörg Helmschrot

**Affiliations:** 1 Institute of Geography, University of Bern, Hallerstrasse 12, 3012 Bern, Switzerland; 2 Géosciences Environnement Toulouse, University of Toulouse, CNRS & IRD, 14 Av Édouard Belin, 31400 Toulouse, France; 3 UFR Sciences de la Terre et des Ressources Minières, Université Félix Houphouët-Boigny, Abidjan-Cocody, Côte d’Ivoire; 4 Directorate of Earth Observation Programmes, EO Science, Applications and Climate Department, European Space Agency (ESA-ESRIN), Largo Galileo Galilei, 1, 00044 Frascati, RM Italy; 5 Mohammed VI Polytechnic University, IWRI, Ben Guerir, Morocco; 6 Stellenbosch University Water Institute, Stellenbosch University, Private Bag X1, Matieland, 7602 South Africa; 7 Institute of Meteorology and Climate Research, Karlsruhe Institute of Technology, Wolfgang-Gaede-Str. 1, 76131 Karlsruhe, Germany

**Keywords:** Earth observation products and services, Transdisciplinary, Societal needs, End-users, Co-production, Collaboration, Community of practice, Africa

## Abstract

Africa stands to gain from Earth Observation (EO) science, products and applications. However, its use and application remain below potential on the continent. This article examines how EO can better serve the needs of African users. First, we argue that a successful uptake of EO services is conditional on understanding the African context and matching EO development and deployment to it. Using reference cases, we find that actors outside Africa drive most EO initiatives, whereas country-level expenditures on EO remain low. Recent developments, such as the African space policy and strategy, and initiatives in partnerships with Africa-based organisations to develop a community of practice on EO hold the potential to fill the identified gaps. The analysis indicates that most EO users are either government organisations or researchers, with very few cases involving other types of users. It is generally assumed that users at the local levels are educated and digitally literate, or that the transmission of EO-based knowledge is achieved by government officers and researchers. Although still very few, potentials are emerging for the private sector to deploy EO products and services such as crop or index-based insurance directly to farmers. These private initiatives have prospects for further developing indigenous EO capacity as envisioned in the African space policy and strategy. We then formulate recommendations for a transdisciplinary approach that integrates user contexts, attributes and needs to enhance the uptake of EO products and services in Africa. We conclude by proposing actions to close some of the identified gaps and seize emerging opportunities.

**Supplementary Information:**

The online version contains supplementary material available at 10.1007/s10712-022-09724-1.

**Article Highlights**

- Africa-based communities of practice - Notable rise in Earth Observation applications and users over the past 2 decades
- Build capacity in Africa for field data provision and technical know-how for validating Earth Observation products
- Multi-level/-sectoral partnerships pivotal for the continued uptake of Earth Observation products and services in Africa
- Low direct connection to local natural resource users with Earth Observation products and services despite potential
- African countries need to invest more in supporting their research organisations through in-country funding schemes

## Introduction

Tremendous progress has been made in access to Earth Observation (EO) in the last decades—in providing EO products, data and services (EOPS). Application areas include land use, natural resources exploration and exploitation, agriculture, hazard monitoring and early warning systems, public health and environment, settlement and migration dynamics.

EO datasets are increasingly in the public domain, thanks to the efforts of the US National Aeronautics and Space Administration (NASA), the French space agency, Centre National d'Etudes Spatiales (CNES), the European Space Agency (ESA), the Japan Aerospace Exploration Agency (JAXA), and the Indian Department of Space Research Organisation (ISRO), among others. In the context of the Copernicus Programme (European Commission 2014, 2021), ESA provides free access to EO data from various European and non-European missions. Thus, for decades, EO data has informed decisions, for example in weather, climate, crop monitoring and early warning systems. Examples are the U.S. Agency for International Development’s Famine Early Warning Systems Network—FEWS NET (Funk et al. 2019), and the Centre régional de formation et d'application en agrométéorologie et hydrologie opérationnelle (AGRHYMET) of the Permanent Interstates Committee for Drought Control in the Sahel (CILSS) (Traore et al. 2014). Some African countries have also invested in developing nano/cube-satellites, (Argoun 2012; Woldai 2020) such as Algeria (AlSat), Nigeria (NigeriaSat) and South Africa (SumbandilaSat, SUNSAT, ZACube-2). Digital Earth Africa, uses freely accessible EO data to provide an increasing number of decision-ready products (Digital Earth Africa 2022).

*Opportunities offered by EOPS are thus widely recognised.* With increasing global interest to monitor terrestrial and marine processes, especially in the context of sustainable development and climate change, the United Nations Geospatial Information Management Initiative (UN-GGIM) developed an Action Plan for Africa from 2016 to 2030 (United Nations. Economic Commission for Africa—UNECA 2017). Equally, *the African Union (AU) has put forward its digitalisation strategy* (African Union 2020a), and African Space Strategy—ASS (African Union 2019a, b) to support its Agenda 2063 that amongst others aims for inclusive growth and sustainable development that is people-driven, and reliant on indigenous potentials (African Union 2015a, b). The ASS strongly advocates increasing Africa's presence in space-borne initiatives (African Union, 2019a, b). It envisions "an African space programme that is user-focused, competitive, efficient and innovative" (African Union 2019a: 13). The establishment and fostering of a high number of communities of practice (CoP) are considered, amongst others, a measure of its success (African Union 2019a). CoP are defined here as the involvement of experts and stakeholders along the EOPS value chain, and include scientists, data providers and a wide range of end-users allowing them to support knowledge and information exchange.

*Despite recognised opportunities, Africa faces various constraints in harnessing the full potential of EOPS.* The ASS acknowledges that "African user needs are not well quantified and documented", there are "inadequate skills in space science", "duplication of efforts and suboptimal coordination", "fragmented space activities, not aligned with continental goals", and the "limited funding …allocated for space science and technology" at continental scale (African Union 2019a: 11). The ASS also identifies "intra-continental partnerships fostering space science collaboration", and "existing and established centres focusing on the exploitation of geospatial data" as the continent`s current strengths.

Yet, *opportunities for EOPS exist where it can serve local needs.* The increasing awareness of its societal benefits by the African public and its contributions to addressing global change challenges offer opportunities for further development and use in Africa (African Union, 2019a: 11). To enhance uptake, the African Union (2019a: 11) regards "international partnerships for co-developing space platforms, EOPS", and "learning from existing satellite programmes to strengthen the continental capacity" as crucial. The African Union (2019a: 11) also recognises threats to realising the ASS such as the "over-reliance on financial and technical support from outside the continent", the inadequacy of national space programmes "to assimilate and adopt rapid technological advancements", and a "lack of a focus on user needs and innovation in delivering relevant space services and products". An uncoordinated “continental approach to multilateral space agreements and guidelines" is a further limitation (African Union, 2019a: 11). To utilise opportunities and address threats, the African Union, (2019a: 12) suggests establishing communities of practice for sharing experiences and best practices, defining user needs, and minimising "the duplication of efforts".

EOPS has widely been applied in African agriculture through early warning, crop insurance and by extension, aiming to improve users` decision-making related to natural resources management strategies, livelihood, food security, and climate change responses (Whitcraft et al. 2019). Although some EOPS have been successful in providing a decision basis for users in African agriculture (e.g. FEWS-NET), EOPS use is largely restricted to implemented applications.

In Africa, *user adoption of digital applications remains low* (CTA 2019). In the area of digitalisation for Agriculture (D4Ag) in Africa, an EOPS-related field, the number of farmers and pastoralists actively using D4Ag applications is low (15–10%) (CTA 2019). CTA noted that D4Ag users in sub-Saharan Africa comprise youths (over 70% of registered users), whereas only 25% are women farmers. Hence the need for better targeting, especially from a gender perspective (CTA 2019). It is thus crucial to ask which end-users are using EOPS and how to enhance uptake. Such a question is important considering the potential contributions of EOPS to addressing the challenges of climate variability and climate change facing Africa and the use of EOPS in the various global to national initiatives (e.g. The UN decade on restoration, the Sustainable Development Goals). Such responses also require engaging actors at multi-levels, from local users, agricultural extension services, local and regional services producers and providers, to policy makers.

Yet, due to the multiple and potential uses of EO data, *many EOPS are often not explicit about the targeted users.* Pictorial representations of users on websites (e.g. farmers) generally do not coincide with the actual users, e.g. practitioners in government ministries or researchers. Often, little or no explanations are provided on whom these “users” or “end-users” are. Knowing EOPS users in Africa will enhance planning, monitoring and evaluation of the effectiveness of EO initiatives for sustainable development (EO4SD), a goal often flagged by many EOPS initiatives. *This paper thus aims to identify EOPS users (end-users) in Africa and make recommendations on adopting a multi-level transdisciplinary approach to enhance user uptake.* Our analysis is illustrated with examples from EO projects and is guided by these specific objectives:

1. Discussing the African EO-related sector concerning the provision and use of EOPS.
2. Identifying the target users and transdisciplinary scope of selected EOPS initiatives in Africa
3. Analysing users’ capabilities and skills to use the provided EOPS and by so doing, highlighting the potentials or limitations of such EOPS to address users’ (societal) needs.
4. Explaining how a multi-level transdisciplinary approach can enhance user adoption of EOPS, and by extension, increase their effectiveness to address societal problems.

Insights from such a multi-level transdisciplinary lens can support EOPS providers, including their funders, to better target users of EOPS in conceptualising, designing, planning, implementing and deploying EOPS in Africa.

## Understanding the African Context of Deploying Earth Observation Products and Services

For EOPS to realise its potential, it needs a conducive environment. EOPS are not standalone but are often a part of a socio-technical infrastructure, comprising the manpower and the relevant skills, the hardware and energy needed for their operation. EOPS also operate within a policy context, which may foster or constrain its operation and use.

First, *policies can foster or be detrimental to EOPS.* Only a few African countries have a national space programme (African Union 2019a). Woldai (2020) discusses the need for such an EO strategy and framework by the African public sector to foster an enabling environment (e.g. space policies, capacity building, participatory approaches, etc.) to benefit public–private partnerships (PPPs) cf. (Kganyago and Mhangara 2019). The ASS thus fills this gap and provides the framework for an African EO programme (African Union 2016; 2018). Adopted in 2016, it serves as a flagship programme of the AU Agenda 2063 in support of promoting science, technology and innovation, investing in human capital development, sustainable natural resources management, effective private and public sector development and the promotion of PPPs as well as innovative resource mobilisation in the continent. The ASS has identified six main objectives, which underpin its ambitions to further develop and strengthen the EO sector in Africa. The objectives are: (1) addressing user needs; (2) accessing space services; (3) developing the regional market; (4) adopting good governance and management; (5) coordinating the African space arena; and, (6) promoting intra-Africa and other international cooperation (African Union 2019b). The ASS and supporting documents thus equip African countries with a policy framework and tools to develop national policies and enable national space agencies to coordinate efforts at the national level.

Second*, infrastructure (e.g. energy, telecommunication, Internet connectivity, *etc*.)* is crucial to developing Africa-based EOPS. Yet, studies show that *access to electricity* in Africa is heterogeneous. For example, based on data for 29 sub-Saharan African countries (excluding South Africa) only about 50% of companies have reliable access to electricity in three countries—Liberia, Namibia, and South Sudan (Blimpo and Cosgrove-Davies 2019). Among households, access is lower in many African countries with less than a third of households in Nigeria, Kenya, Mali, and Tanzania having access to reliable electricity; in contrast, 80% of households have access most of the time in only six countries, Cabo Verde, South Africa, Eswatini, Gabon, Côte d’Ivoire, and Mali (Blimpo and Cosgrove-Davies 2019). Where electricity is available, energy cost is high, which represents a significant fraction of monthly expenses for most households and firms. Power outages also cause firms to perform below expectation (Cole et al. 2018). An implication for EO data analysis and use is that the basis for powering computers, including access to the Internet, cloud-computing services, data centres and power to recharge cell phones remain inadequate. *While the Internet (e.g. 4G, and fast optic-fibre network) and smartphone penetration in Africa* have been increasing in recent years, reducing the barriers to reach users and accessing the Internet at a reasonable cost, their use is still constrained by access to electricity. Telecommunication services now provide access to the Internet in many African countries, including in rural areas via 3G/4G networks, but internet bandwidth remains heterogeneous, which poses a further barrier.

Third, *information systems and technological innovations, through improved geo-Information technologies have enhanced access to new types of EO data* and provided access to analytical tools for processing spatial big data up to planetary scales. Although personal computers have also become more affordable, high-performance computers are still out of the reach of many African entities. Only 6 out of 54 African countries run EO receiving and tracking stations (Woldai 2020). ArcGIS^®^/QGIS and other open-source GIS and remote sensing software and cloud-computing platforms dedicated to EO such as Google Earth Engine (GEE) and the Amazon Web Services (AWS) have also stimulated interest in EO and Geo-information technologies in Africa (Woldai 2020). This is also demonstrated by the development and operation of open-source portals as part of the Global Monitoring for Environment and Security, and Africa (GMES and Africa) Programme (see below for more details on GMES and Africa). One example is the Wetland Monitoring and Assessment Service for Transboundary Basins in Southern Africa (WeMAST) Portal providing EO-based wetland information to basin commissions (Southern African Science Service Centre for Climate Change and Adaptive Land Management 2018a, b). Another example is the portal supporting the Marine and Coastal Areas Management in western Africa (GMES and Africa 2019).

Fourth, *adequately trained human capacity and job opportunities are critical to sustaining EOPS in Africa.* Do African countries have adequate human capacity and skilled professionals to analyse EO data and operate EOPS? It is also about the need for academic institutions that build capacity in "EO, engineering, technology and data analysis" (Woldai 2020: 109) and a thriving private sector "as an engine of economic growth" (Woldai 2020: 109). Comparing end-user categories in public and private sectors with the academic sector, the *majority of EO activities in Africa are conducted through university education, training* and research activities at national and regional levels (Woldai 2020). Major achievements in the development of EO and Geo-information in Africa in the public sector, relied on the *"free and cost-effective access to EO data"* such as provided by the ESA, NASA, the CNES and the European Commission (Copernicus). International and regional organisations, as well as development partners, have provided data, service and training. The high proportion of African EO professionals trained outside Africa attests to the capacity-building role of international scientific organisations and development partners (Woldai 2020).

Fifth, *most local universities and research institutions are underfunded in Africa.* Strengthening local universities, research institutions and the private sector will further increase manpower in Africa. Yet, regional inequalities exist in terms of capabilities in higher education with *critical gaps in capacity building in EOPS persisting* between Western, Eastern, Northern, and Southern Africa. Research organisations and universities face various challenges: trained capacities remain inadequate, curriculum and infrastructure in many African universities remain outdated and Internet connectivity remains inadequate such as for downloading satellite data. Cloud computing (e.g. Google Earth Engine, GEE) remains a good option for low Internet connection, and limited local data storage capacity, but underfunding which creates difficulties for academic institutions to provide Internet access to their staff and students, is an obstacle to the more widespread use of Internet-based services for research and learning (Kigotho 2021). A decline in the quality of graduate training, mainly due to declining public investments in higher education and research, translates to inadequate research capacity in many countries in SSA (Fuglie et al. 2020). This situation is further compounded by the constraints posed recently by the COVID-19 pandemic, including the reallocation of available funds from other sectors toward public health (CABRI—The African Cooperative Initiative on Fiscal Reform 2022). Success in increasing local content in learning materials and the number of indigenously trained scientists and engineers demand the necessary political commitments (Prakash et al. 2020; Woldai 2020; Kigotho 2021).

## Africa's Use of Earth Observation Products and Services

**Fig. 1:**
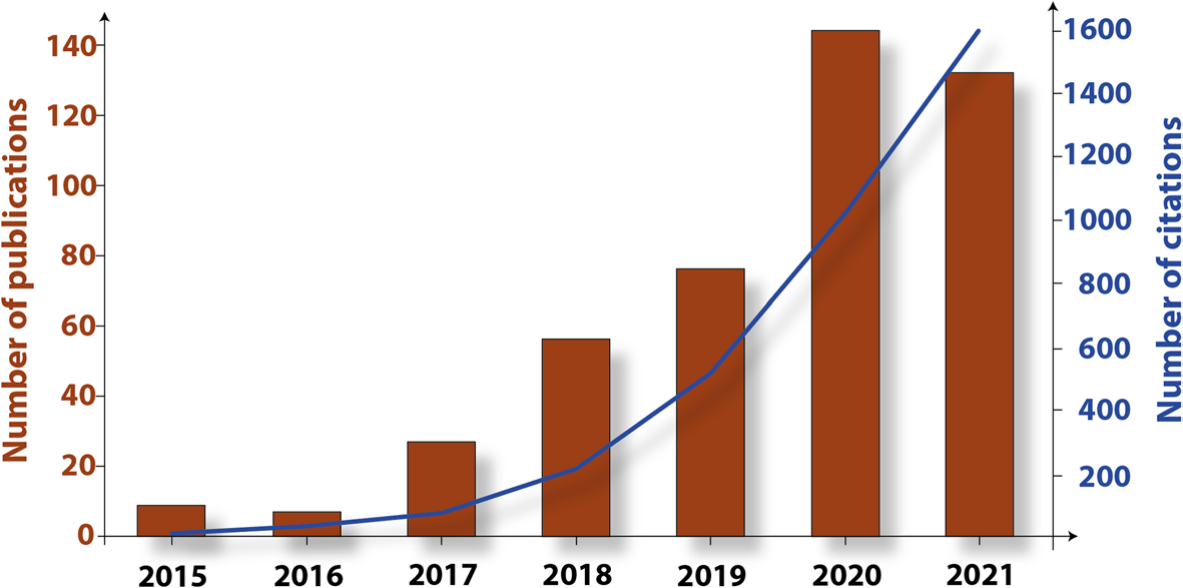
Publications on African countries using Sentinel-1 or Sentinel-2 satellite images

**Fig. 2:**
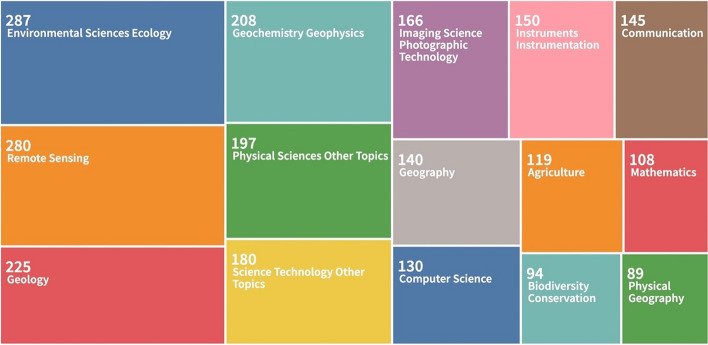
Scientific subjects of the publications on African countries using Sentinel-1 or Sentinel-2 satellite images (the numbers refer to the number of publications classified in each research area, according to Web of Science, one publication may be classified in several areas of research)

*Research on Africa using EOPS continues to increase.* The results of the bibliometric survey we conducted on the Web of Science (see supplementary file) on the use of ESA Sentinel-1 and Sentinel-2 products for research on Africa (2015–2021), yielded in total, 450 publications (Fig. 1). There is a continuous increase in the number of publications on African cases. Figure 2 shows the breakdown of subject areas utilising Sentinel images on the African research cases. Our finding reveals that the majority of usage is in the Environmental Sciences, Ecology and Earth Sciences (e.g. Geology, Geophysics and Geochemistry).

*Spatial differentiation of EOPS applications shows most EO activities have been at the macro-level* (international, continental, regional and national) involving international and development organisations such as the NASA/CNES/ESA, UNECA, FAO; UNEP, FEWS NET, Africa regional centres, African Union, EU, UN-SPIDER, Group on Earth Observations, Collaborative Research Programs, multilateral and bilateral aid projects. This finding buttresses the fact that although publications utilising EOPS in an African context are increasing, these publications are not necessarily led by researchers in Africa-based research institutions or universities. However, as shown by similar studies related to climate change, there is an increase in first and co-authorship originating from the continent (Kapuka et al. 2022). This is also in line with the findings of Kumar and Mutanga (2018), which identified very few GEE-based studies from developing countries, in particular, even lesser on Africa or by Africa-based researchers. This reflects the limited access to the technology and inadequate human capability despite that the EO datasets are freely available.

*Further, a major flow of EOPS goes to public sector organisations* but a lack of high-resolution (typically better than 10 m/pixel) data limits the applications, for instance for urban planning (Andreu et al. 2019; Bhargava et al. 2018; Mahabir et al. 2018; Prakash et al. 2020; Weerasinghe et al. 2020; Wimberly et al. 2021; Martin 2022). When the needed EO data is not freely available, the cost of acquiring such datasets becomes another major barrier to access and use (Dube et al. 2016). The flow of EO to the public sector at local levels has been very low, probably because of the inadequate technical capacity of local-level actors (e.g. local government officers) to use EO data. *End-users of EOPS beyond the government entities at local levels* are hard to find. Yet for EO to contribute effectively to grassroots development, engaging local-level actors such as agricultural extension officers, forestry and environmental officers, land resource users, local NGOs and farmers is important. The question is whether they can digest and use EO and even when they do, whether they have access to the infrastructure underpinning the use of EO. Co-designing EOPS is thus important to meet such needs of local actors as their motivations are taken into account in the process (Adelle and Lightfoot 2018).

Thus, many approaches to EO use in Africa have been *top-down, technology-driven*, largely stemming from research partnerships that are often with a limited spatial scale and short-term project focus (Bégué et al. 2020). In contrast, a few long-term projects, such as those conducted under the Global Monitoring for Environment and Security and Africa (GMES and Africa) exist (Azumah et al. 2021; Lavaysse et al. 2021). Generally, *weight is given to the macro-*levels (national and regional) rather than to the micro-levels. Emphasis is on strategic monitoring issues at macro-levels and even less on the everyday challenges of sustainable land management, sustainable livelihoods and/or hazard risk reduction at the local level. *Most end users are either trained professionals, in research and university networks* such as members of the African Association of Remote Sensing of the Environment (AARSE 2019), employees of government ministries, departments and agencies, parastatal companies, and very few in the private sector.

*National-level systematic EO-based monitoring is few.* While some countries have remote sensing data-based biomass (crops and/or fodder) forecasting systems, e.g. South Africa, (Bernardi et al. 2016); Senegal (Diouf et al. 2015); Morocco (Centre Royal de Télédétection Spatiale 2021), *few extend to local land users.* Examples include EO-based mobile phone applications to pastoralists in Mali and Burkina Faso (SNV 2019), monthly agrometeorology bulletin and 3-months rainfall forecast for farmer groups in Botswana (Department of Meteorological Services 2021) or EO supported Index-based drought risk financing (IBDRF) targeting pastoralists mainly in East African drylands (Fava and Vrieling 2021).

A remarkable proportion of African land users are *poor and lack the economic and other resources to pay for the costs* of operational EO-based decision support systems. A challenge faced by many providers and implementers at national levels is the lack of budgets and difficulties to establish structures filling such gaps. This is partially explained by the lack of national planning priorities in the sector but also the lack of support to develop respective infrastructure and capacities for enhanced EO and EOPS use. Hence knowing who the users of EOPS are will help to consider options for contributing to operational and institutional costs in the long-term by international donors (Sibiko et al. 2018; SNV, 2019).

In terms of the African private sector in EO, Woldai’s (2020) surveys of 2016 and 2020 show that of the 229 companies surveyed across Africa, 130 are involved in EO and Geo-information sciences across 28 of the 54 African countries, with key markets segments ranging from local and regional planning, environmental monitoring, pollution, climate, agriculture, geology and mining. In West Africa, it is worth noting the case of the Geomatica company based in Senegal (Geomatica 2022), and Geomatics Nigeria Ltd (Geomatics Nigeria 2022), which provide various training and services.

Most businesses are in *collaborations with international companies*, NGOs, universities, private companies and foreign governments including space and aid agencies as key sources of funding, geographic data, information, training and support (Woldai 2020: 102). Yet challenges faced include a lack of development funding/venture capital even when they have business ideas, unfavourable policy and legal provisions and lack of information on space-based activities, applications, analysis of the demands and markets among the citizenry (Woldai 2020).

Although the private sector runs ground stations, provides tracking services, consultancies and works on space infrastructure, *knowledge and usage about these African satellites are very limited due to a lack of clarity on access to images acquired*. While 11 African countries launched satellites in the period 1998–2020, information about these satellites and scientific publications are uncommon, although many of them are active in earth observation, scientific experiments, and communication, with a few in military reconnaissance and climate change and weather monitoring (Woldai 2020).

*Despite their African labels,* the African countries and their launched satellites are generally *dependent on partnerships with key actors outside Africa*, e.g. Surrey Satellite Technology owned by Airbus building the satellite for Algeria (Surrey Satellite Technology Ltd 2016). Ghana Sat 1, for instance, was designed and built in conjunction with the Kyushu Institute of Technology Birds-1 program, which has the goal of helping countries build their first satellite (Ministry of Communications and Digitalization 2017). *Are African national space agencies then providers or end-users?* Studies by Woldai (2020) reveal that although African space agencies perform in both roles, they are barely involved in developing satellite technologies. In some cases, they build partnerships with satellite building companies from the Global North (e.g. from European Countries, Russia, or China). Such partners provide technical training programs for African space engineers for the operational use of satellite receiving and tracking stations. Here, the agencies act both as receivers of data and as providers of products developed after utilising these data. An exception is South Africa where South African universities have been involved in constructing a satellite. The majority of African countries remain mainly as receivers of EO technologies.

Thus, *awareness of and access to Africa-generated EO remains low.* There is little information about local-level actors in the field of technology provision in Africa. Despite the African satellites, one can conclude that African satellite data, except for South Africa and Morocco, currently plays no substantial role, neither in the private business sector (Woldai 2020) nor to our knowledge in the use of such data in academia. Further, *the sharing of EO datasets remains non-existent between agencies and researchers, and between African space agencies* thereby stifling partnerships between African countries (Woldai 2020). Although a Nigeria satellite had the best capture of Hurricane Katharina, a lack of cooperation and *weak government support of private sector actors stifles innovation,* and restrictive bureaucracy for obtaining EO data makes users prefer to download Sentinel or Landsat data for use instead (Woldai 2020).

Bégué et al. (2020), in their review of existing remote sensing products and services for public decision making, show that *inadequate capacity building* is a persisting challenge in enhancing user adoption of remote sensing products. Analysing the gaps between remote sensing science and relevant data needed for agricultural policies (public sector/government), they conclude that the existing EO initiatives are inadequate to address the needs of governments for better agricultural and land management and planning (Bégué et al. 2020).

Increasingly, satellite data availability for the future is no longer a major issue as the flood of satellite data from the Copernicus and Landsat programmes provides adequate data but the question remains *whether and how the EO data, products and services are being used effectively*. Bégué et al. (2020) argue that international donors have supported remote sensing projects only in a few African countries and even then, most of the projects are driven by technological questions rather than by developmental needs (technology-driven approach) hence not *conducive to evolving into independent initiatives that draw on local expertise and resources.*

*Recent years have seen an increasing number of activities and funds* addressing EOPS directed at Africa. Initiatives and programmes such as GMES and Africa (see below) are indicators highlighting the efforts to further increase EOPS development and deployment in Africa. Here, various research projects develop EOPS that are applied in smaller contexts (cf. van Zijl 2019). These are in addition to those EOPS from international cooperation hence raising the concern of *duplication of activities* (G20 Agriculture Ministers 2011). Building on what already exists and drawing on lessons learned can thus reduce the duplication of efforts (UNECA 2017; Becker-Reshef et al. 2020).

*Achieving long-term sustainability of EOPS thus remains a challenge in Africa,* from various perspectives. These include the inadequate local capacity and training, and the corresponding inadequate institutional capacity to absorb trained professionals, a small unconsolidated African community of EO-practice, inadequate funding, lack of viable business models, a dependence on inputs from the Global North, and duplication of actions, among others (UNECA 2017; Saah et al. 2019). EO contributes to Agriculture 4.0 (Agriculture 4.0 is an evolution of precision agriculture making use of digital technologies to process and analyse in real-time relevant data), but its prospects in Africa are constrained by inadequate and/or a lack of knowledge, poor literacy skills, limited finance, and infrastructure (Jellason et al. 2021). These limitations contrast with the need to create jobs, reduce poverty and contribute to sustainable natural resources management in Africa and the promises that EOPS hold for addressing these.

The above constellations and achievements but also limitations call for a better consideration of how to enhance user uptake of EOPS in African countries and the key roles of African governments.

## The Role and Commitment of African Governments and Actors

*The role and commitment of African governments* to increase investments in providing adequate infrastructure for technology uptake, especially in agriculture and natural resources management is key to enhancing awareness and the use of EOPS (Kigotho 2021). A major African policy strategy to improve agricultural development is the Comprehensive Africa Agriculture Development Programme (CAADP) in which African governments committed to using at least 10% of their total budget for agriculture by 2025 (Maputo/Malabo declaration- African Union 2003). However, in 2017, only 20 out of the 47 reporting African Union (AU) countries were on track to meet the 2025 commitments while in 2019, only four out of the 49 reporting AU countries remained on track to meet the 2025 commitments (African Union 2019c, 2020b). Benin (2018) analysing data from 25 African countries from 2001 to 2014 reports the CAADP has had a remarkable positive impact on spending in agricultural expenditure by government, and development assistance for agriculture, land and labour efficiency. However, the author highlights that with time, the impact of CAADP on government agricultural spending wanes, indicating a substitution effect between government funding and those from external sources for agriculture (Benin 2018).

Thus, *investments in agriculture and the environment by African governments remain generally low.* Growth in expenditures on agricultural research and development among half of the African countries is either zero or negative (Fuglie et al. 2020: 23). Fuglie et al. (2019) reveal that African governments need to address the acute shortfall in research spending, and the inadequate motivation of the private sector to invest in technology and innovations. For Sub-Saharan Africa (SSA), the trend of the ratio of research and development (R&D) to gross domestic product (GDP) between 1981 and 2011 declined, while the ratio of R&D/Cropland is US$9 per hectare, the lowest in the world (Fuglie et al. 2019). It thus becomes clear that EOPS cannot thrive and reach its full potential in such an environment.

The *investments of the private sector in Agricultural R&D are heterogeneous,* with South Africa showing a high private sector share of 19% relative to other African countries in 2008 (e.g. Kenya, Senegal, Tanzania, and Zambia had 8% in 2008). Often, cloudiness restricts the use of old EO datasets hence, some EO data from long-term archives in ESA/NASA/NOAA cannot be used. Considering the drawback of limited spatial data such as agricultural masks, crop calendars and meteorological data (Fritz et al. 2019) and missing data for historical analysis, also due to the lack of satellite receiving stations in much of Africa pre-Sentinel times, business cases may be developed to provide ancillary data. Such data could be in the form of digitised historical aerial photographs in African archives, measurements from ground stations, in particular in the wake of restricted movement due to the COVID-I9 pandemic,—a potential business opportunity for small entrepreneurs.

The above elaborations show a context of limited support for research and development by the African public sector, with some improvements in recent years but also a context that remains largely constraining for the EOPS to achieve its full potential. In the following, we draw from reference cases, selected based on available information, to identify patterns of EOPS applications and potentials for enhancing user uptake in Africa.

## Reference Cases of Earth Observation Products and Services and their Uses Across Africa

*Facilitating the access to and use of EO in Africa is part of the strategy of several organisations from the most industrialised parts of the world.* Several publicly funded or private initiatives have been launched over the last decades and are presented below. Although EO data products are primarily developed for the Global North (Bégué et al. 2020; Tonneau et al. 2019), various international initiatives provide free access to ready-to-use EOPS through web portals. They include, for example, the National Aeronautics and Space Administration’s Moderate Resolution Imaging Spectroradiometer (NASA MODIS) Land Team, the European Commission's Joint Research Centre (JRC), the European Organisation for the Exploitation of Meteorological Satellites (EUMETSAT), and the European Space Agency (ESA) and the European Copernicus initiative.

Although many African countries and their research or public institutions still face low bandwidth, power outages and limited infrastructure for data download and storage, globally, the launch of new satellites, with higher resolution, and high frequency of observations has led to an exponential increase in data, which has *resulted in a paradigm shift*. Data do not need any more to be downloaded by the user to their local machines for further processing; hence, cloud-computing services have flourished over the last decade. Again, here, some services are funded with public money, whereas others are private and offer researchers worldwide data and tools for EO and mapping. These services potentially reduce the consequences of the different levels of computer infrastructure between Africa and the rest of the world and facilitate capacity building. We focus first on public initiatives and then present a few private initiatives that have a significant impact in Africa. These initiatives have been selected to cover categories we deem insightful for enhancing user uptake (public and private initiatives driven from outside and within Africa), to reflect the scope of initiatives, but do not capture all past or ongoing initiatives using EOPS. Other international research funding bodies (other than UN, EU and US initiatives) also provide remarkable support to EO-research in Africa.

### Public Partnerships with Africa for Earth Observation—United Nations Organisations

Many illustrative cases of international cooperation with United Nations Organisations abound. Here we draw on the case of the Food and Agriculture Organisation (FAO) Desert Locust Information Service (DLIS), established in 1978. An early warning system that uses among others satellite imagery for responding to locusts in Africa and Asia (FAO 2016). Here the forecasts and advisories produced are sent to national governments and donors for their subsequent actions.

Desert Locust (*Schistocerca gregaria*) and other locust species outbreaks affect the semi-arid and arid regions of Africa, the Near East and South-West Asia, thereby threatening food security in these regions. The FAO DLIS engages with national governments who send FAO data on locust incidences. With the increasing availability of satellite data, global positioning systems (GPS) and geographic information systems (GIS) (FAO 2022a, b), the FAO has enhanced the timeliness and accuracy of data used in the DLIS (FAO 2016). EOPS-related products play a key role in DLIS—through the eLocust3, a data recording and transmission system based on computer tablets. Field staff in 19 vulnerable countries, collect data from remote locations "and transfer it in real-time via satellite from the field to their national locust centres (national geographic information systems), before transmission to the DLIS at the FAO Headquarters in Rome". (FAO 2016: 2). The FAO then conducts an integrative analysis of the locust data "with weather and habitat data and satellite imagery" to assess the situation and provide forecasts (FAO 2021, 2022c; Ellenburg et al. 2021). The FAO conducts field assessments, builds national capacity, and coordinates DLIS operations and emergency assistance during locust outbreaks. Further, it provides monthly bulletins on locust situations for each affected country. In training national staff on the eLocust3, the FAO adopted *a training of trainers approach* whereby it trained the national locust information officer who then trained staff on the use of the application (FAO 2016). End-users "were staff of the national locust control centres in plant protection departments within the Ministry of Agriculture of each locust-affected country" (FAO 2016).

With the locust infestation of East Africa in 2020, the Penn State University, collaborating with the FAO, developed a mobile phone version based on *crowdsourced* locust observation data, the eLocust3m, which can be downloaded from App stores (FAO 2022a; b; Tabar et al., 2021). "The United States National Oceanic and Atmospheric Administration (NOAA) – which jointly operates weather satellites with NASA", "is using FAO’s latest data from East Africa to expand a model that projects locust flight paths". (FAO 2020). Satellite data from the NASA and the European Space Agency (ESA), provide FAO experts information on rainfall, soil moisture and vegetation conditions and by extension information on locations with potentially favourable breeding conditions to anticipate the locust hazard (FAO 2020).

*Challenges amongst others include building and maintaining a community of practice* to ensure locus officers could maintain their knowledge over periods without locust outbreaks, maintaining collaboration with software developer partners and the willingness of the affected countries to pay for data transfer service (FAO 2016). Success is reflected in the degree to which these challenges have been addressed.

*A success factor amongst others is maintaining open standards and using open-source software* in such an application (FAO 2016). The FAO reports that *long-term public–private partnerships and commitments* were key to the success of the eLocust3. Such PPPs, range from free 3D extension of Novacom Services-eLocust3 software (eLocust3 3D is a custom open source map application) from the Italian company Trigolis Srl., free World Wind technology from the United States NASA and free services on supplied hardware by Panasonic^®^. *Country commitments* through annual contributions and covering costs for training through the FAO Regional Desert Locust Commissions as well as *data sharing* ensured the sustainability of the use of the eLocust3. Further, *"collaboration with users, developers and vendors from the outset"* and throughout the project and follow-up during operationalisation have been important for the success achieved in locust monitoring (FAO 2016).

### Public Partnerships with Africa for Earth Observation—the European Union

The African-European partnership for the digital transformation of Africa is part of the strategy of the European Commission with Africa, with the European Space Agency (ESA) as a key actor. ESA has indeed played and continues to play an important role in *offering raw data, products, tools and capacity building to facilitate the EO uptake in Africa.* The ESA TIGER initiative, founded in 2002 (ESA 2022a)—2004 started a consultation process in collaboration with African water authorities, technical centres and other stakeholders in both the water and the EO sectors in Africa. The initiative was reinforced with training activities aiming to prepare African researchers to fully exploit the increasing observation capacity, including the constellation of Copernicus Sentinel satellites.

The EO Africa Project (African Framework for Research, Innovation, Communities and Applications, funded by the ESA) aims to *foster an African-European R&D partnership*, to facilitate the adoption of EO and related space technology in Africa. It follows *an African user-driven approach* with a long-term vision for the digital era in Africa—via an R&D Grant provided through Invitation to Tenders (ITTs, ESA 2022b). It builds on 17 years of experience gained through the ESA TIGER initiative (ESA 2022a) and its African network of EO experts. Together with the framework of EO Africa, the advent of the European Copernicus programme and observations collected by its Sentinel satellites offer unprecedented open access and free data for African institutions, amounting to 1.2 petabytes per year over the African continent.

The Sentinel-1 (radar) and -2 (multi-spectral) missions provide geospatial information (radar data and multi-spectral images with 13 bands in the visible and near-infrared domain) at resolutions relevant to various areas of fundamental and applied research. They include, for example, geology/exploration of natural resources, oceanography, hydrology, agriculture, monitoring and mitigation of natural hazards, and monitoring of the degradation of the environment due to anthropic activities, including the consequences of climate change (Phiri et al. 2020; Torres et al. 2012). The frequency of this data acquisition over the same place (5 days with the combination of Sentinel-2A and -2B) is a decisive advantage when compared to previous image datasets. It enables rapid evaluation of critical situations and even emergency management through specific programs of data acquisition (e.g. Copernicus Emergency Management Service mapping component, or the European Union’s GMES Initial Operations—Emergency Management Service (GIO-EMS), ESA 2022c; European Commission 2022c; Kucera et al. 2013). It also allows the construction of long-term time series, which are needed to monitor the effects of human activities such as artisanal mining in West Africa, cf. (Ngom et al. 2020).

*The frequency of acquisition of Sentinel EOPS* and the use of all-weather radar data products of Sentinel-1A and -1B has *largely addressed the cloud coverage constraints* in tropical regions, that cause lack of data during the rainy seasons, for example in West or Central Africa. Cloud computing, such as the Google Earth Engine platform, is now also widely considered an alternative approach to data download and processing at local institutions (Azumah et al. 2021; Kumar and Mutanga 2018). In this respect, the Network of Resources (NoR) is another ESA initiative to facilitate the use of cloud environments by users. The NoR Discovery Portal (ESA 2019) presents a portfolio of innovative operational platforms and cloud services operated by actors from participating countries available through the NoR. The Discovery Portal is also the gateway to request NoR sponsorship providing an estimate of the cost of services including the ceiling level of sponsorship generally awarded by ESA for non-ESA projects.

The European Union Pan-African Global Monitoring for Environment and Security (GMES) and Africa Support Programme (GMES and Africa) (European Commission 2015; African Union 2017; European Union 2015a, b) builds on long-term cooperation between the EU and the African Union. The GMES and Africa aims to improve the decision-making capacities of African policy makers and planners through the provision and use of EOPS to design, implement and monitor policies and to enhance sustainable management of natural resources (European Commission 2015; African Union 2017; European Union 2015a, b) for the private, public and academic sectors. Acknowledging the limitations of past numerous EO initiatives in Africa and their short durations, the GMES and Africa initiative builds on infrastructure and capacity established by previous initiatives and is active in various areas. The GMES and Africa aims at the  following major achievements : (1) Maintain, improve and sustain EO access in Africa; (2) Inform African policy makers and users by providing an African Water and Natural Resources service, and a Marine and Coastal monitoring Service; (3) Enhance capacities of African public institutions and private sector to use EO-based information at regional and national levels; (4) Inform African policy makers, administrators, entrepreneurs, scientists and civil society of opportunities provided by EO data and geospatial information technologies at regional, national and local levels (summarised from European Union 2015b: 17–21; African Union 2017).

The GMES and Africa acknowledges the *critical role of end-users in the development and implementation* of EO-based services and products as a key indicator for successful EOPS applications. The GMES and Africa is thus based on the principle to *involve end-users in the definition of products, constantly liaise with the users* during the programme development as well as the development and implementation of training programmes for end-users (African Union Commission 2018). Through *stakeholder analysis,* the GMES and Africa identified users and their needs at the political and technical levels, private users and other end-users, but *only directly targets African policy-makers and planners at national, regional and continental levels*.

To strengthen research and teaching capacities, the GMES and Africa Initiative established a network of Academia in 2019 (GMES and Africa 2021a, b). Through dialogue with the African network of universities, the GMES and Africa initiative identified training and capacity needs for academia, *adopting an approach of training trainers and building on what already exists,* thereby aiming "to strengthen the capacity of African academic institutions". Through a GMES and Africa Academic Network (GAAN), the GMES and Africa Initiative also aims to involve GAAN institutions in its training and research activities as key stakeholders for programme sustainability. Such an approach promises to "foster *collaborations and cross-fertilisation* among universities and create synergies with other projects and programmes", “establish different working groups and sharing resources and reports "through the Virtual Network Platform", and operationalise the Distance Learning Platform by uploading and making over 100 courses freely available (Saley 2021). GMES and Africa also offers a Copernicus Masters in Digital Earth led by the University of Salzburg, Austria—"Erasmus Mundus Joint Master Degree" (Lang 2021).

As of 2020, the GMES and Africa initiative made progress in various dimensions to improve access to EO data including receiving stations, upgrading existing software and digital applications, and enhancing access to Copernicus data. Such developments include, amongst others, a Regional Flood Event Database and an android-based mobile application for reporting and assessing heavy flooding (MIFMASS), an ocean forecasting and tools for tracking accidents at sea (MARCOSOUTH), apps to facilitate navigation on the Congo River (CICOS), and for automatic reception and processing of spatial data (various projects). Others include providing access to baseline data for wetland managers for assessing wetlands dynamics, enabling the protection of livelihoods for wetland dependent communities (WEMAST), monitoring service for forest activities (SEFAC) or the installation of satellite receiving and processing stations in various countries for meteorological and environmental applications (various projects and partners), the monitoring and assessment of seasonal crop and rangeland conditions in East and Central Africa (GDZHAO), monitoring fishing zones for the Mediterranean Sea (NAfCOAST), or an integrated vessel tracking tool for monitoring illegal fishing and smuggling in the Southern African Development Community (SADC) region (MARCOSOUTH) (Space in Africa 2022a).

In the course of the GMES and Africa Project in North Africa (Algeria, Egypt, Libya, Morocco, Mauritania and Tunisia), *the need “for more effective integration of user needs” in developing* EO-services was identified (Observatoire du Sahara et du Sahel—OSS 2018). To improve the integration of user needs into the development of EO-services, national-level studies and workshops have been conducted, for example by the OSS (the Sahara and Sahel Observatory) and its partners in Algeria (OSS 2019a), Morocco (OSS 2019b) and Tunisia (OSS 2019c). While activities are reported, only limited information is provided on the findings or the impacts of the GMES and Africa Project in the region.

At the project level, multi-faceted training and capacity-building activities have been undertaken. The recently launched GMES and Africa Digital Learning Platform (DLP) aims to improve "skills and expertise in Earth EO applications in Africa". with "target beneficiaries being experts in the field, and early career youth. With over 103 courses on EO and almost 600 registered users, the GMES and Africa project achieved its training objectives for 2020". The GMES and Africa has compiled success stories in a video to highlight how and to what extent it has been beneficial, and its impacts on end-users and beneficiaries (Space in Africa 2022a). The consortia involved in the GMES and Africa increased their training portfolio in 2020, "through the DLP and use of open-source software. More Masters and PhD students have been sponsored, internship opportunities created, and networks formed with African universities to expose them to knowledge sharing. *Engaging and raising the awareness of beneficiaries and end-users* have led to an uptake of digital communications tools" (Space in Africa 2022a).

*Partnerships between projects* are actively sought, for example, training Tunisian technical staff in the fields of agriculture, agricultural research and remote sensing was conducted in the frame of both the AfriCultuReS and GMES-Africa projects by the OSS and its Tunisian partners (OSS 2020) or exchanging lessons-learned in the projects (OSS 2020).

The GMES and Africa Initiative plans to *involve the private sector,* and by providing free and open-source software, integrating "ICT with EO-leveraging on cloud computing capabilities" as *a way to address the infrastructure and electric power limitations.* For example, the project WEMAST, implemented by a SASSCAL-led consortium (Southern African Science Service Centre for Climate Change and Adaptive Land Management 2018a, b) involved the private sector during the development, deployment and operation of the WEMAST geoportal, which provides data and products related to wetland monitoring and assessment to stakeholders in the SADC region.

An assessment of the progress and achievements of the currently awarded projects shows that the projects have reached different levels of implementation with some projects having developed user-demanded and operational EO-based services and others having focused on training and user needs assessment. Despite these different levels, the GMES and Africa Initiative, through its projects has made notable contributions to fostering EO applications and training in Africa.

### Public Partnerships with Africa for Earth Observation – United States of America

The world, including in particular Africa, continues to gain from research and development initiatives using the NASA/USGS (United States Geological Survey) Landsat Program, which has the longest continuous freely available EO record of the Earth's terrestrial surfaces (NASA 2022). This long-term record builds the basis for various EOPS initiatives.

The SERVIR (not an acronym but Spanish word to “serve”, emphasising the notion of services) is a joint development initiative between NASA and the U.S. Agency for International Development (USAID). "SERVIR is a global network of five regional hubs in Asia, Africa (Regional Centre for Mapping of Resources for Development in Nairobi, Kenya, the Agrometeorology, Hydrology and Meteorology (AGRHYMET) Regional Centre in Niamey, Niger), and the Americas, supported in partnership with the USAID and NASA’s Capacity Building Program in the Earth Science Division’s Applied Sciences Program" (Mayer et al. 2020).

SERVIR East and Southern Africa works across Africa (SERVIR GLOBAL 2022). SERVIR *provides local decision-makers with the tools, training and services* needed for action on climate-sensitive issues such as disasters, agricultural security, water management and land use. Since the mid-2000s, SERVIR has worked with over 250 institutions, including local and national government agencies, universities, non-governmental organisations and other specialised groups. SERVIR offers custom-tools development, in-person and online training at all levels to facilitate the use and integration of EOPS into existing programs. SERVIR generally focuses on disaster management, ecological forecasting, agriculture, public health, air quality and water resources management. Most training within SERVIR is offered via a partnership with the ARSET program (Applied Remove Sensing Training Program), with dedicated online resources.

The GEOGLAM Crop Monitor for Early Warning—the Group on EO Global Agricultural Monitoring (GEOGLAM) Initiative, a part of NASA harvest projects run by the University of Maryland, USA and an international G20-agriculture ministers endorsed programme (G20 Agriculture Ministers 2017), also has a remarkable focus on Africa. It also aims at the increased use of EO to enhance decision-making, actions and policy on food security and agriculture (Nasaharvest 2022; Becker-Reshef et al. 2020).

GEOGLAM is *collaborative and builds on existing initiatives* "through coordinated Earth observation, capacity development, monitoring, and research and development activities" thereby synthesising existing knowledge and establishing a "GEO Agriculture Monitoring *Community of Practice* (Ag CoP)". The GEOGLAM Crop Monitor for Market Information (AMIS) and crop monitor for early warning CM4EW) are two major services. Becker-Reshef et al. (2020) acknowledge *"considerable geographic overlap among early warning systems"*. The GEOGLAM thus *grew out of requests by international development and aid donors* to international organisations to link AMIS and existing global, regional or national early warning systems (EWS) for food security and vulnerability to a synthesised joint early warning information (Becker-Reshef et al. 2020). Such EWS include for example the. FAO-GIEWS-Global Information and Early Warning System, the USAID-FEWS-NET—US Agency for International Development—Famine Early Warning Systems Network and the WFP-VAM-World Food Programme's Vulnerability Analysis and Mapping) (G20 Agriculture Ministers 2011; Becker-Reshef et al. 2020). The CM4EW aims to enable early response and avert food crises.

National and local level EWS are thus equally important and besides the *demand for harmonisation from international donors*, each country and each context has its specific place-based contexts that justify its "own" early warning systems. Together "with national ministries in Tanzania, Uganda and Kenya, the GEOGLAM CM4EW has recently been adapted for national contexts (Becker-Reshef et al. 2020)". International, regional, and national crop monitoring organisations have co-developed the CM4EW, (USAID-Agrilinks 2022) using EO data thereby demonstrating EOPS’ value in an operational setting for supporting food security decisions (Becker-Reshef et al. 2020). Becker-Reshef et al. (2020: 2) argue that "the CM4EW is a community activity" that *builds on existing resources* as the involved organisations have common goals, share data, information, networks, and experiences, use common definitions and crop monitoring criteria, and through monthly deliberations, build consensus on crop conditions globally. Such a collaborative approach improves the quality of the information provided to decision makers by reducing ambiguities, filling data gaps and communicating assessments with one voice (Becker-Reshef et al. 2020).

The GEOGLAM CM4EW focuses on countries facing food insecurity risk, mostly countries of the Global-South, with a prominent Africa focus. It links to the Crop monitor for the G20 agricultural Market Information System (AMIS) for *global commodity crops* such as wheat, maize, soybeans and rice, both endorsed by the G20 agriculture ministers (G20 Agriculture Ministers 2011). Becker-Reshef et al. (2020) report that the logic for developing the GEOGLAM CM4EW is that EO-based early warning on crop conditions would inform *objective, timely information to the markets thereby reducing food price hikes.*

Becker-Reshef et al. (2020: 2–3) report that "the CM4EW builds largely on the existing regional and global-scale crop/agricultural monitoring systems operated by the *main agencies mandated to assess crop conditions and production worldwide* within the context of early warning and food security. They include the FAO GIEWS, (FAO 2022d), the FEWS NET Data Portal (FEWS NET 2022), the WFP VAM, the China CropWatch from the Chinese Academy of Sciences' Institute of Remote Sensing and Digital Earth (CAS RADI), the Crop Explorer from the US Department of Agriculture (USDA 2022), and the JRC Monitoring Agricultural ResourceS (MARS) system (European Commission—Joint Research Centre 2021; Becker-Reshef et al. 2020).

The CM4EW provides a common platform that links these existing international platforms, thus providing a *basis for dialogue and consensus* on crop conditions necessary to trigger early warning, thereby creating a *community of practice at a global level.* The CM4EW bulletins provide monthly information on crop conditions at the subnational scale based on integrating assessments from regional analysis of the partner organisations in a process coordinated "by the GEOGLAM Crop Monitor Coordination team at the University of Maryland", for the GEOGLAM Secretariat in Switzerland.

Of the above-mentioned EWS, the FEWS NET has a long history, providing EO-based early warning and analysis on acute food insecurity in West, East and Southern Africa (FEWS NET 2022). Supported by NOAA and USGS and with strong engagement of local partners, FEWSNET develops real-time agroclimatic early warning information products and services, which help to alert on emerging or likely food-related disasters. As a principle, data and products are made available to African stakeholders to support decision-making. In addition, training programmes are regularly conducted to improve local and national capacities in the fields of remote sensing, modelling and in-situ observation.

### An African Regional Initiative: Hazards Watch for East Africa

Besides its activities in climate prediction, the Intergovernmental Authority on Drought and Development (IGAD) Climate Prediction and Applications Centre (ICPAC) is an East African regional partner to the CM4EW. Through the CM4EW, the ICPAC links to other existing platforms such as the USAID famine early warning system that has been operating in the region for at least the last 20 years. Further, its ICPAC East Africa hazards watch developed by an interdisciplinary team, monitors climate variability and change including floods and droughts, pests, desert locusts and associated crop failures to improve access to risk information for the region (ICPAC 2022a). Decision support information is provided via emails and mobile phones. According to ICPAC, the hazards watch built on open source bases, is "*the first public African system of its kind, … developed in Africa for Africa* that is continuously improved based on user feedback and multiple data sources" (ICPAC 2022a). The integrated data layers enable viewers to comprehend the changes in temperatures and rainfall over the last century (ICPAC 2022a). The ICPAC also provides a monthly-quarterly East Africa crop monitor for the region (ICPAC 2022b), which users can sign-up for or download. Besides these products, the ICPAC provides weekly, monthly and seasonal weather forecasts as well as climate monitoring and is involved in the regional Food Security and Nutrition Working Group. Through advisories, it informs decision-makers about ensuing conditions that might warrant their action.

### Earth Observation Tools among the Private Sector in Africa

The private sector is active across Africa in providing EOPS, although not as prominent as public sector actors. *EOPS underpins various private-sector initiatives on weather index insurance* cf. (Fava and Vrieling 2021). For example, the Swiss Re® supports the Africa Risk Capacity (ARC) and smaller index-based weather insurance in Africa. Swiss Re, Doreo Partners, an impact investment firm, and its agricultural franchise Babban Gona, established the first weather index insurance programme in 2014. Based on satellite data to determine the lack of rainfall, the programme pays out automatically to smallholder farmers, thereby protecting them against the risk of adverse weather patterns (Swiss Re 2015).

The Agriculture and Climate Risk Enterprise Ltd. (ACRE) is a registered insurance surveyor in Kenya, an insurance agent in Rwanda and in Tanzania, with projects in Uganda, Ghana, Malawi, Senegal, and Mozambique. It evolved from the Kilimo Salama project (established 2009), which was funded by the Syngenta Foundation and the Global Index Insurance Facility (GIIF)—a multi-donor trust fund financed by the European Union, Japan and the Netherlands, and implemented by International Finance Corporation (IFC) and the World Bank (ACRE Africa 2020; Syngenta 2016). ACRE provides weather indexed insurance to farmers, based partly on EO data (rainfall from weather satellites, soil moisture, and vegetation index) and a multiple crop cover (maize, beans, wheat, sorghum, millet, soybeans, sunflowers, coffee, and potatoes). ACRE also draws on *public–private partnerships* with "different funders and partners to reach smallholder farmers". The funders include "Innovate UK, World Bank, Alliance for a Green Revolution in Africa (AGRA), International Development Research Centre (IDRC) and Australian Centre for International Agriculture Research (ACIAR) ". In 2014, it was projected to "reach 3 million farmers across 10 countries in Eastern and Southern Africa by 2018" (ACRE 2014). Farmers register with ACRE via a USSD code and interact via their mobile phones and can make and receive pay-outs via scratch cards at affordable amounts and MPESA (a mobile-phone-based payment platform of Safaricom^®^ mobile phone company in East Africa). Village agents of ACRE (village champions) equipped with smartphones collect farm profiles using the “see it grow” app at all stages of crop growth and remit the pictures to agronomists for monitoring (ACRE 2021).

*Partnerships between philanthropy, private equity and public sector actors* have driven such investments. The Syngenta Foundation is a majority shareholder of ACRE, and ACRE receives investments from the Lundin Foundation, Grameen Crédit Agricole Foundation and LGT Venture Philanthropy, and grants from the International Finance Corporation of the World Bank Group (IFC), international development aid and other sources (Syngenta 2016). Greatrex et al. (2015) report that by 2013, ACRE had covered "almost 200,000 farmers in Kenya, Rwanda, and Tanzania, with an insured sum of 12.3 million US dollars, whereas the insurance pay-out was USD 370,405". The Climate-Smart Agriculture Guide (2022) drawing on the IFC and World Bank (2014a, b) states that "insured farmers invested 19% more and earned 16% more than their uninsured neighbours and 97% of the farmers insured in 2013 received loans linked to the insurance". Although ACRE has made tremendous progress in reaching smallholder farmers, Sibiko et al. (2018) using choice experiment data from Kenya, highlight the need to provide farmers with relevant rainfall measurements and thresholds regularly to increase transparency and farmers’ willingness to pay for weather index insurance.

*EOPS are beneficial for agricultural advisory services, which are unevenly distributed across Africa.* Ethiopia provides about half of the extension capacity of sub-Saharan Africa (Fuglie et al. 2020). In Africa, the extension officer-farmer ratio is very high and e-extension and mobile phones could reduce the costs of outreach to farmers (Bachewe et al. 2018; CTA 2019). Improving value chains will likely attract more diverse actors such as agribusinesses, and provide opportunities for NGOs to disseminate information (Fuglie et al. 2020). The potential for using EOPS in local extension services has thus not been widely exploited.

The African Union Agenda 2063 aims at *increasing indigenous initiatives in EOPS.* Geomatica (Geomatica 2022), a company based in Senegal that specialised in geomatics solutions, addresses this gap. Geomatica comprises a group of experts in GIS, Remote Sensing, Cartography, Computer Science, Web-mapping and Topography, and offers solutions for setting up databases, processing geographic information and distributing geographic data on several platforms. It offers a *package of solutions adapted to the management of local communities* such as land parcels, topographic plans, urban networks, and land tenure.

### Other Regional, National or Local Initiatives for Earth Observation in Africa

Over the last decades, the popular Google Earth Observations have been widely used as a generic tool in various research areas. African researchers and other African actors in EO also *increasingly use the Google Earth Engine, the publicly available cloud computing service,* offering access to large volumes of EO data. It requires skills in programming language, and remote sensing processing techniques. It also offers various statistical tools and includes packages in the artificial intelligence domain. The (heavy) computation time is achieved on the platform and only results (not raw data products) may be downloaded from the server at the end of the processing step. Naturally, this private free service comes with the usual risks of interruption or modifications of the economic model (change of conditions of use from free services to paid services). Google Earth Engine favoured in any case the development of capacity in remote sensing techniques in Africa. During the 2015–2021 period, about 75 articles were published (with co-authors based in African institutions based on analysis using the GEE, and 2/3 of these research articles were published over the last two years. Thus, indicating the increased interest in African research for this platform (source: Web of Science). Most areas of research concerned by these studies include geology, agriculture, biodiversity, meteorology and atmospheric science, and environmental sciences.

Among other notable initiatives, the Digital Earth Africa—DE Africa (Digital Earth Africa 2022) is a recently launched platform funded by a US-based charitable trust and the Australian government (Lewis 2020). DE Africa offers the Digital Earth Africa Map, which is a website for map-based interactions. The provided tools allow exploring DE data, products, and visualising the African continent with satellite images to understand its geographic diversity and changes through time. The DE Africa Sandbox is a cloud-based computational platform that operates through a Jupyter Lab environment. It provides a limited, but free compute resource for technical users and data scientists to explore DE Africa data and products. It enables access to remote-sensing data and analysis tools for ad-hoc report generation and the rapid development of new algorithms. This analysis environment is improved continuously to meet the needs of users. Connected to DE and supported by a *public–private partnership* (e.g. ESRI^®^, African countries) the Africa GeoPortal is an open mapping community of local GIS professionals from various African countries, working together to provide data and insights across Africa (Africa Geoportal/ESRI^®^ 2022).

The Geophysics Research Group for Europe and Africa (GIRGEA 2022) aims to develop Space Physics in African countries within the framework of the United Nations for Basic Space Science Initiative. Currently, the main theme of the research is Space Meteorology. The work of GIRCEA, therefore, focuses on many space-based applications, including GPS, satellite communication and their perturbations in relation to space weather. The African Initiative for Planetary and Space Sciences (AFIPS) (AFIPS 2022) was to build an African network of researchers in the domain of space and planetary science. It currently has about 300 members in Africa, and two workshops were organised under the AFIPS umbrella—one in Kenya about space weather, and a more general one in Ethiopia focusing on EO (Baratoux et al. 2017). This initiative also highlights the synergy in the development of sensors between Earth observations and the exploration of the Solar System, and the interdisciplinary research and training activities that can be achieved in this domain.

*EOPS also inform decision-making at national levels.* For example, Botswana uses EO in its annual assessment of vulnerability to drought and household food security. The first stage of this assessment involves the EO-based analysis of drought. Indicators analysed with satellite images include cumulative rainfall and rainfall anomaly, changes in vegetation such as the Normalised Difference Vegetation Index (NDVI) anomaly and Vegetation Condition Index (VCI), which capture agricultural drought, rangeland and wildland fire (burnt areas). The EO outputs serve as the basis for subsequent field assessments during the mid-growing season in Botswana (Rural Development Council 2019). The assessment outcomes then form the basis for the Government of Botswana declaring a drought year with the launch of corresponding relief programmes, thus linking to local levels.

In the field of water management, there is an enormous potential *to link satellite data with citizen science to improve the assessment and monitoring* of water levels in streams, precipitation, and groundwater. As the technical aspects of using satellite EO for hydrological modelling are improving (Walker et al. 2019), and more technologies exist such as cameras, web-based mapping, and navigation systems, there are more examples of citizens providing field data that can be directly used to improve water resources management (Buytaert et al. 2014). For example, in Kenya, residents are sending text messages containing their visual observations of the water level in their local streams to researchers who then use that data to improve models that ultimately improve water allocation (Weeser et al. 2018). Although this example does not directly involve satellite EO, the potential for the three-way synergy between citizen science, satellite data, and computing and communication technology is obvious. In this example, participants were very enthusiastic, sending text messages costing 1 US cent. Researchers did find that paying a small reward or at least a reimbursement increased the participation rate by seven. Simple cell phones that could send text messages were a reliable enough data source for instantaneous water level monitoring. Motivation for participation stemmed directly from a local susceptibility to floods and droughts and a clear understanding that data can help mitigate those problems. However, that relationship must be reinforced by regular feedback between the research and the citizen scientists. Other examples of citizen science improving water resources management exist in Tanzania, South Africa and Ethiopia (Njue et al. 2019).

The water accounting plus framework is an example of how a hydrologic model, informed mainly by EO data, can be run within a management context, for example, that of the Volta Basin and provide clear policy recommendations (Dembélé 2020). The need for clear information such as this one may help achieve that goal—delivery of consolidated information to policy-makers rather than contradicting results or messages. Furthermore, a further step of this project could include some citizen-based ground observations that would further refine the interpretation of the satellite data and the implementation of the model. The final, optimistic goal, in this case, would be for the model to provide deliverables that would directly improve grassroots water management and supply information to small-scale land users and owners. Such information could be for irrigation or for recommending soil moisture management, or even early warning data regarding rainfall and/or upcoming dry spells and droughts (Foster et al. 2020; van Ginkel and Biradar 2021).

Various countries have developed land use maps, e.g. some West African countries (Institut national de l’information géographique et forestière 2022), using EO to support decision-making, e.g. the South African National Space Agency (SANSA) (Kganyago and Mhangara 2019). However, it is mainly national and sub-national organs such as government departments and agencies and research organisations and universities that use such data and platforms (Bégué et al. 2020). EOPS thus largely remain at the national and sub-national levels with limited reach and use at the local levels and by local land users.

## Identifying and Characterising Users of Earth Observation in Africa

**Fig. 3:**
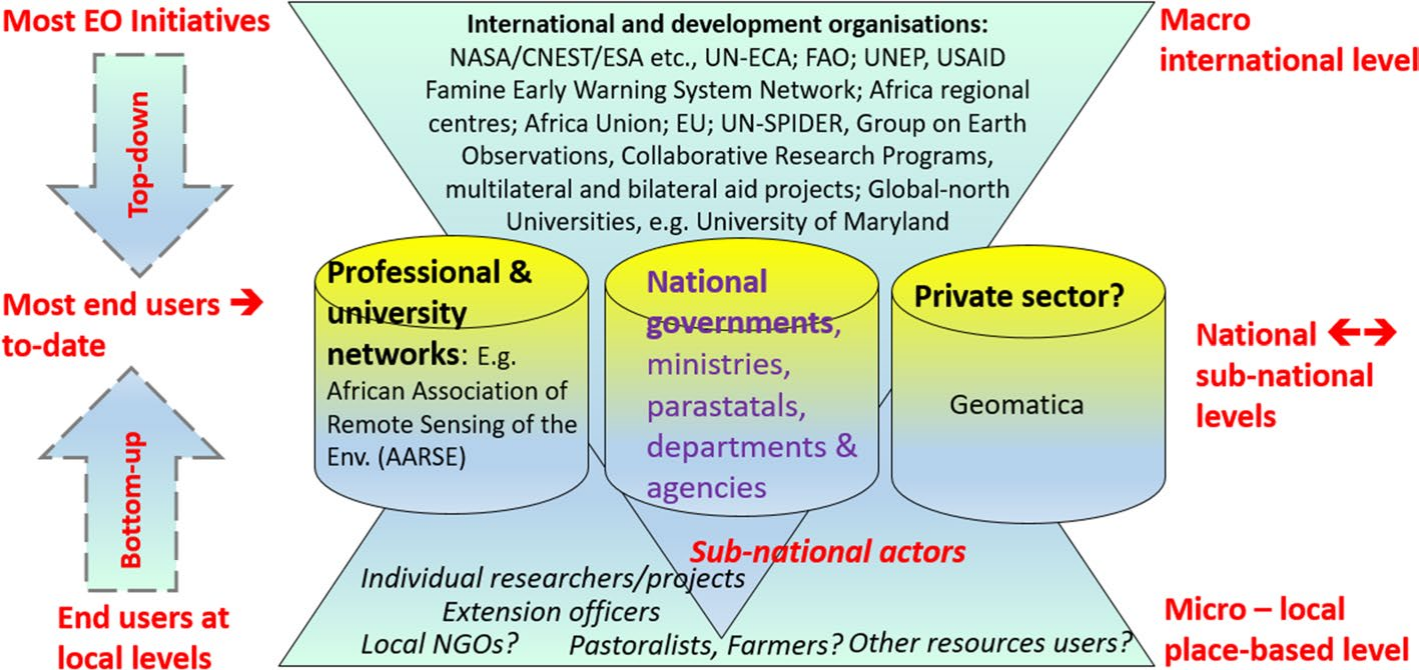
Identified categories of providers and users of EOPS in Africa (derived from literature, e.g. Woldai 2020; Becker-Reshef et al. 2020, and programme websites)

From the above reference cases, different providers and end-users can be identified. As Fig. 3 shows, *the main providers are located outside the African continent* but work in partnership with the African Union or with African regional organisations or national governments through bilateral cooperation. The end-users are the public sector, including government ministries, departments and agencies, mainly at national levels, African research organisations and universities and rarely individuals such as farmers, pastoralists or agricultural extension officers at local levels.

The reference cases show that *most focus of EOPS initiatives is at the continental, regional or national level* with most users in the national public sector, research and academia. Little is said about the business sector or the land resources users and their support structures such as the agricultural advisory services. Our interpretation of these initiatives is that they assume that governments and research actors will disseminate their knowledge to the local levels. This reflects a *top-down approach*, with its associated benefits and drawbacks. In most cases, there is no follow-up about knowledge transmission, nor feedback, except at the same levels among peer-professionals.

While the practice-relevance of EOPS has been proven in various studies when users of EO are mentioned, *a certain level of education and skill is implicitly assumed of a certain type of user* that has the technological infrastructure and the relevant skills to use the generated products and services to address needs. Barro and Lee (2013) show that in 2010, an average adult worker in Sub-Saharan Africa attended formal schooling on average for five years with women having lesser years of formal schooling on average, hence reflecting also a significant gender gap in EOPS use.

*Everyone is a potential user of EOPS*, which for instance includes a simple examination of the weather forecast and satellite images. Each citizen can benefit from EO science. To understand better the EO institutional landscape in Africa, the African Union Commission is surveying African institutions to inform the operations of the African Space Agency (Space in Africa 2022b). As end-users of EO products and services are as diverse as are the EOPS themselves, there is a need to know first the users, align EOPS deployment to their education levels and map the different types of users to the various types of EO products and services. Doing so will enable identifying target users and better measure progress in enhancing their uptake of EOPS. In the following, we discuss from a transdisciplinary lens, lessons from the reference cases for enhancing user uptake of EOPS.

## A Multi-level Transdisciplinary Integration for Enhanced Uptake of Earth Observation Products and Services in Africa

*A transdisciplinary approach has the potential to account adequately for the multi-level processes and the multiple actors involved in EOPS* (Fig. 3). Such a transdisciplinary approach entails deriving EOPS through integrating both societal needs and perspectives with scientific perspectives of the issues an EOPS aims to address through (1) collaborative problem framing, (2) co-producing solution oriented knowledge and (3) applying the co-produced EOPS in science and societal practice (Lang et al. 2012; Bergmann and Jahn 2017; Pohl et al. 2017). The expectation is to enhance the relevance of the EOPS for societal practice. Transdisciplinary sustainability research has been proposed as an approach to integrate non-academic actors into the research process as a way to combine diverse knowledge, experiences, and multiple values thereby *creating a community of practice,* accounting for actors’ needs, values and preferences and creating ownership of problems and identified solution options (Lang et al. 2012). Hence, for EOPS to be sustainable, involving (expected) users is necessary.

First, a key question is *to what extent problems addressed by EOPS are collaboratively framed between EOPS providers and end-users* and to what extent do EOPS providers know the end-users and end-user requirements (stakeholder mapping)? The assumption is that if end-users are involved in co-defining the EOPS and the problems they tackle, users’ interests, needs, experiences and skills can be better integrated at all stages of the EOPS process and help build a joint understanding of the EOPS, hence improving the prospect of achieving expected outcomes (Lang et al. 2012). The reference cases show that EOPS providers *partly know the end-users but only at a very high macro level,* among high-level policy-makers (governments to governments) but rarely to the researcher-community of practice or local resource users in Africa. Hence, the end-user in such cases are often government ministries, African regional organisations or development cooperation actors with certain mandates for monitoring (e.g. ICPAC, AGRHYMET, FEWSNET).

Second, drawing on the reference cases, we discuss how early in the process of designing and deploying EOPS collaboration is initiated. The assumption here is that it is best to involve the end-users (however defined) already in the planning stages of an EOPS or as early as possible and not assume that users will apply the EOPS once designed and developed. Depending on the type of EO applications, there might be arguments for later interaction with, or integration of, end-users. While end-user involvement may not always be feasible (e.g. in conflict contexts), at least, EOPS aimed at end-users needs to build on an adequate understanding of the needs and expectations of those societal actors so the EOPS can be better targeted at those end-users (e.g. considering appropriate form and language). The reference cases are not clear in this dimension—often, decisions again are made at the high policy level, sometimes between the African Union and the European Union or even at the G20 donor-level (e.g. GEOGLAM) and it is then left to the continental commissions to translate to the regional and national levels, with the locals levels often ignored. *Thus a top-down and supply-driven approach to EOPS predominates.* Thereby EOPS reproduce power asymmetries—as knowledge is a form of power if EOPS are accessible to a minority relative to the broad mass of anticipated end-users that can potentially benefit, it is likely to cement or create new power asymmetries, which is not conducive for EO4SD.

Third, the reference cases show *inadequate attention paid to the intellectual (e.g. digital literacy) and material capabilities* (e.g. steady energy and Internet access) required for effective and sustained EOPS use. Issues related to EOPS use within Africa are framed without considering the limitations (e.g. power outages, weak computing infrastructure despite cloud computing options) of the African context. As the reference cases show, *most EOPS in Africa are external to the continent* hence, for dependency to be reduced, African governments need to *commit more to EOPS than they currently do*. While skills development is progressing, deficit infrastructure constrains trained capacity.

A further question is to *what extent are lessons learned in designing and applying the EOPS re-integrated into scientific and societal (user-) EOPS practice?* The reference cases show that participants in EOPS arena in Africa and the world have *realised the multiple initiatives and duplication of EOPS initiatives in Africa*, at least in the public sector. This realisation in part triggered the AMIS and crop monitor for early warning CM4EW to *build on what is existing and thereby build a COP* that links the various EOPS actors in African EWS. Such a COP has reduced duplication of efforts, hence freeing up resources for use elsewhere. Yet, the duplication remains and further efforts are required to consolidate communities of practice in the various sectors in which EOPS initiatives are active in Africa. Through partnerships, for instance, the GEOGLAM and the GMES and Africa initiatives engage with African scientists and practitioners, hence *building or enhancing existing communities of practice in EOPS.* At the national level, reduction in duplications may be possible by the elaboration of national space policies, via institutions or agencies in charge of coordinating these activities at the national level (e.g. national authorities on remote sensing, or national space agencies).

The reference cases show that *developing and deploying EOPS is a multi-level and multi-dimensional process involving various actors.* EO is by nature a non-contact, proxy-based collection of data about the object of investigation. An observation across distance connotes that certain aspects of the observed, for instance, land cover and land use, require ancillary data (e.g. direct field measurements and ground-truthing), which is often place-based, to complement or validate EO data. EOPS is thus multi-dimensional and requires not only expertise in satellite remote sensing analysis and interpretation but also knowledge about local ecosystems from ground-truthing). Hence, it is crucial to incorporate available knowledge to enhance the uptake and ownership of EOPS. Thus, collaboration between space- and remote sensing science, and non-academic actors, including local-land users is likely to *increase the legitimacy of identified solution options, and raise ownership and accountability* (Lang et al. 2012). Further, the *acquisition of such ancillary data also represents opportunities for capacity building and research in EO techniques,* via the use of field sensors that are necessary to validate and understand space-based observations (e.g. multi-spectral or hyperspectral imagery requires the use of field portable hyperspectral instruments).

Within countries, various national level actors such as from national ministries of environment and agriculture, research and academic organisations are involved in EOPS, thus, capturing its multi-level dimension. However, the rather constraining circumstances and the different national economic conditions discussed in Sect. 2 of this review, remain a challenge to overcome. The picture at the local grassroots and individual actor levels is even more diffuse. Hence, these multi-levels and the various involved actors and their interests need adequate consideration. *A transdisciplinary approach* thus *enables deliberating on which actors to involve, their relevant capacities and roles* in the different phases of EOPS development (Muhar and Penker 2018).

The EO data suppliers need to first understand the user needs in context before any success of data uptake can be achieved. *It is not enough to provide EO products if the infrastructure is inexistent.* However, optimistic developments include the emergence of local companies that provide in-situ data but these companies should not be reduced to providing in-situ data only; this is an emerging opportunity to contribute to remote sensing efforts, which could lead to a profitable business.

While many reference cases are driven by their expectations to contribute to improving human wellbeing, they are also driven by research goals that do not put the citizens at the forefront and are not widespread. Yet, the potential to *bring EO directly into citizen science for community empowerment* has already been demonstrated (Walker et al. 2021). In Ethiopia, 2 million farmers are sending their pictures to CIMMYT and get a warning of a disease but such initiatives need to be scaled up (e.g. using the approach of seeds of a good Anthropocene—good examples, Hartkamp 2001). Further, *open street map communities* exist across Africa, which gathers young people that are using satellite imagery to delineate data (Humanitarian OpenStreetMap Team, HOT 2021). This is a "lighthouse" effort, demonstrating the potential for grassroots widespread data assimilation and utility. Such efforts need to be supported by training and be included in calls for projects, and as start-up for the private sector with potential contributions to job creation. Although open street map communities have been growing, they are still not ubiquitous. HOT (2021) claims that individual membership, as opposed to chapter membership, and local leadership would build the organisation. Additionally, awareness of its existence, accessibility, and utility would expand its use. The *transparency delivered by satellite data needs to be handled with care*—e.g. drones (UAV: Unmanned Aerial Vehicles), or high-resolution satellite imagery, may be perceived as a violation of privacy, or sacred forests, hence deliberations with local key actors are necessary for their mapping (Arthur et al. 2020; Ceperley et al. 2010). While the use of EOPS or technologies such as UAVs may create employment, there is a need for formal and ethical guidelines on how to manage sensitive findings from UAVs or high-resolution satellite imagery. While early warning system alerts help to identify areas with biomass resources for grazing and surface water for swiftly moving livestock, providing such information firsts needs to be within the frame of local-national land use regulations to avoid conflicts between land users, highlighting again *the need for EOPS providers to engage with local social-ecological realities.Engagement with EOPS users also means an improved communication of who those users are to the wider public.* Pictures depicting EO applications on websites visited often show local land users either at the forefront with EO products or services depicted in the background (e.g. USAID-Agrilinks 2022). This framing communicates a message of local land users being the direct targets/users of EOPS, which contrasts reports and studies consulted for this study, which show that the targeted end-users are mainly actors at the international, regional and national levels. Local land users may be indirectly targeted if, and when users at the national levels extend or apply the skills and knowledge, they gained to address local level land and resources management challenges. Unfortunately, *no studies exist to show how the "trickling down" to local resource use levels and associated improvements in sustainable land and resources management have been achieved.* Pictures transmitted by these platforms tend to portray local resource users as "beneficiaries", while the actual users (e.g. officers sitting at national ministries, research organisations and universities) are not depicted. Not depicting the actual EOPS users in EOPS communications to the wider public and portraying local resource users as “beneficiaries” reflect the top-down nature of most EO4SD applications.

So, *what kind of users do information and dissemination tools such as newsletters from EOPS applications target?*—A user that can navigate the platform, read automated emails and mobile phone alerts. How many African farmers have the knowledge and skills to assimilate and use the provided information? Better still, which users in the agricultural value chain and environmental management have the capacities and skills to use the provided information? If most African farmers cannot digest this information, then one must look up to the next level of actors that often interact with farmers, that is, the extension services. However, the agricultural extension officers (e.g. crops, livestock, fisheries and forestry) in many African countries, while having the basic education to enable them to access such platforms and information are chronically understaffed and under-resourced. Hence, if EO is to contribute to sustainable development and better inform local resource use and management, these challenges, outside the EO domain need addressing.

Thus, enhancing the use of EOPS for sustainable land and natural resources management *needs a deeper dive into local resource use and management*. However, most of the initiatives do not explicitly focus on this level and implicitly assume that by focusing on national levels, EO use and benefits will *trickle down to local* land and natural resources management. Acknowledging the progress made so far in considering transdisciplinary integration at national, regional and international levels, it is important for future initiatives and research to *assess to what extent national level initiatives made an impact on the ground, and how sustainable these impacts are*. Insights won through analysing the social ecology and political economy of EOPS from a multi-level perspective, can help unearth barriers to EO-uptake at local land and resource management levels and will help achieve the goals of sustainable land and natural resources management. While Whitcraft et al. (2019) argue for *"leaving no pixel behind"*, we add that *"leaving no users behind"* is equally important as EOPS is also about the people—after all, it is those local users who also play a key role on the conditions of the pixels.

The reference cases reveal the need *to balance individual—with institutional capacity building* to create enabling ecosystems for EO4SD. This is crucial considering that many challenges to enhancing the use and application of EOPS are outside the domain of EO—in the wider context of the African political ecology and political economy. Auerswald and Lokesh (2017), and Bégué et al. (2020: 7) thus argue for an economic ecosystem approach comprising three components: "(1) access to images, data and tools; (2) training and capacity building; (3) national/regional strategies." Beyond knowledge, there is a need for creating an "ecosystem" that supports the local generation of knowledge and uptake. Such an approach may help adapt the structure or organisation of EOPS deployment. Often the products and services are *developed outside Africa and deployed to the continent for expected adoption and use, which may initially work but fail in the long term.* One argument brought to address this shortfall has been capacity building. While capacity building has been ongoing for over 30 years, there is increasingly a need to differentiate between individual capacity building and institutional (organisation) capacity building that constructs an "ecosystem" that absorbs trained local professionals into a "functioning ecosystem" that can maintain the EOPS once the external partners depart. This is a well-known challenge in development research and practice that needs to be integrated into EO4SD. Currently, many trained individuals cannot be absorbed by African institutions forcing such trained individuals to obtain employment elsewhere outside Africa, or in other sectors in Africa, hence defying the goal of EO4SD.

Finally, the reference cases highlight a potential thriving EOPS private sector. As in the public sector, *partnerships will remain crucial.* Among other structural factors, power outages and the limited access to good research/business infrastructure such as high-performance computing constrain the application and use of Earth Observation in Africa. These emerging opportunities for private sector engagement also offer potential for providing employment opportunities but require that African governments and civil society *create a context that enables such EOPS businesses to thrive.*

## Conclusions

In this paper, we examined the prevalence of transdisciplinary approaches in reference cases of EOPS and explored the potential for enhanced uptake of EOPS by end-users when a transdisciplinary approach is adopted. We identified various achievements in EO application and use and in capacity building in Africa thanks to local initiatives and international cooperation and support. However, EOPS development and deployment need to understand the African contexts and consider that societal needs vary across these contexts, users and scales. Societal needs are multi-dimensional—development aspirations and commitments, job creation, poverty reduction and development challenges mean that contexts can foster or constrain the use of EOPS. Most reference cases of EOPS show a supply-driven approach with little consultation or active involvement of end-users, who might be varied depending on how end-users are defined. This definition is often lacking, as many EOPS initiatives do not explicitly identify who their target or end-users are. There is also a need for more collaborative initiatives beyond research and at subnational and local levels—a few recent EOPS initiatives are now building communities of practice, a key aspect of transdisciplinarity. However, data on the transdisciplinary processes that EOPS initiatives might have undergone are scarce to inexistent.

In preparing this review, we have had to depend on secondary material as empirical studies are lacking. There is thus a need for in-depth field studies of the various dimensions covered in this review. Although the data and information basis is limited, our transdisciplinary assessment yields various insights: In the phase of *collaborative problem framing*, we conclude from available information and data that among the reference cases assessed, this occurs mainly between international providers and national level users of EOPS. At the stage of *co-producing solution-oriented knowledge*, the collaboration also largely remains at the international-national level with the circumstances of data scarcity compelling providers and end-users at the national level to engage with local users to crowd-source data but not necessarily for the local-level users to become direct end-users of EOPS. *Applying co-produced knowledge in scientific and societal practice* again remains at the national and international levels with an exception of the private sector actors that directly engage with local users (e.g. pastoralists and farmers). There is thus a risk that EOPS remains exclusive to national and international levels but we also find potential for private sector initiatives that directly engage place-based local users thereby bridging this divide. A transdisciplinary approach that focuses on the collaborative process in addition to supply-driven approaches can thus enhance EO application and use. With increasing awareness of EOPS providers of the need to enhance engagement with their end-users, it is expected that actors involved in EOPS initiatives will more and more make such data and information explicit in their documentation hence providing a more solid basis for future studies of transdisciplinarity in the Earth observation domain.

## Supplementary Information

Below is the link to the electronic supplementary material.

Supplementary file1 (DOCX 16 kb)
