# Supplementary material for: Enhancing the Uptake of Earth Observation Products and Services in Africa Through a Multi-level Transdisciplinary Approach
Source: Surv Geophys. 2022 Aug 23;44(1):7–41. doi: 10.1007/s10712-022-09724-1 (PMC9398042; doi:10.1007/s10712-022-09724-1)
Supplement: Supplementary file 1 — Supplementary file1 (DOCX 16 kb) [file 10712_2022_9724_MOESM1_ESM.docx]

**Supplementary file**

**Enhancing the uptake of earth observation products and services in Africa through a multi-level transdisciplinary approach**

**Web of Science bibliometric search**

We conducted a bibliometric survey on web of science, with the following request: AB=("Sentinel-1" or "Sentinel-2" or "Sentinel 1" or "Sentinel 2") AND AD=(Algeria OR Angola OR Benin OR Botswana OR Burkina Faso OR Burundi OR Cabo Verde OR Cameroon OR Central African Republic OR Chad OR Comoros OR Congo OR Democratic Republic of Congo OR Côte d’Ivoire OR Ivory Coast OR Djibouti OR Egypt OR Guinea OR Guinea-Bissau OR Equatorial Guinea OR Eritrea OR Eswatini OR Ethiopia OR Gabon OR Gambia OR Ghana OR Kenya OR Lesotho OR Liberia OR Libya OR Madagascar OR Malawi OR Mali OR Mauritania OR Mauritius OR Morocco OR Mozambique OR Namibia OR NigerOR Nigeria OR Rwanda OR Sao Tome and Principe OR Senegal OR Seychelles OR Sierra* OR Somalia OR South Africa OR Sudan OR South Sudan OR Tanzania OR Togo OR Tunisia OR Uganda OR Zambia OR Zimbabwe) and PY = (2015-2021)
